# Light interaction in sapphire/MgF_2_/Al triple-layer omnidirectional reflectors in AlGaN-based near ultraviolet light-emitting diodes

**DOI:** 10.1038/srep09717

**Published:** 2015-05-26

**Authors:** Keon Hwa Lee, Yong-Tae Moon, June-O Song, Joon Seop Kwak

**Affiliations:** 1 Department of LED Business, LG Innotek Company, Ltd., Paju 413-901, Korea; 2 Department of Printed Electronics Engineering, Sunchon National University, Jeonnam 540-742, Korea

## Abstract

This study examined systematically the mechanism of light interaction in the sapphire/MgF_2_/Al triple-layer omnidirectional reflectors (ODR) and its effects on the light output power in near ultraviolet light emitting diodes (NUV-LEDs) with the ODR. The light output power of NUV-LEDs with the triple-layer ODR structure increased with decreasing surface roughness of the sapphire backside in the ODR. Theoretical modeling of the roughened surface suggests that the dependence of the reflectance of the triple-layer ODR structure on the surface roughness can be attributed mainly to light absorption by the Al nano-structures and the trapping of scattered light in the MgF_2_ layer. Furthermore, the ray tracing simulation based upon the theoretical modeling showed good agreement with the measured reflectance of the ODR structure in diffuse mode.

AlGaN-based LEDs in the near-ultraviolet (NUV) range are attractive for photo-catalytic deodorizing in air conditioners and refrigerators as well as in identifying counterfeit banknotes^1^. In particular, color-rendering due to the high conversion efficiencies of typical phosphors in the UV spectrum has attracted considerable great interest for fabricating white LEDs with AlGaN-based NUV-LED chips for white lighting^2^,^3^,^4^,^5^. On the other hand, AlGaN-based UV-LEDs normally suffer from very low external quantum efficiency (EQE) due to the poor light extraction efficiency caused by the epitaxial absorption of UV light. In addition, reduced indium compositional fluctuations in InGaN quantum wells with a small indium content weakens the carrier localization in localized energy states, which in turn increases the possibility of carrier trapping at the nonradiative recombination centers by the increase in in-plane carrier diffusion. To overcome these obstacles in AlGaN-based UV-LEDs, noble device structures, such as chip-shaped LED design^6^, photonic crystal^7^, distributed Bragg reflector (DBR)^8^, omnidirectional reflector (ODR)^9^, surface texturing reducing internal light reflection, and surface plasmon^10^,^11^, have been proposed.

In particular, ODR, such as Ag- and Al-based triple-layer schemes at the backside of LED chip, has been used to increase light extraction. On the other hand, the reflectance of an Ag-based reflector decreases rapidly in the UV region. Therefore, Al-based reflectors are preferred because of the relatively high reflectivity, wide stop band, and omnidirectional reflection characteristics in the UV wavelength range. The refractive index of a dielectric layer in the ODR structure should be as low as possible. MgF_2_ films, which have a low refractive index in the range, 1.34–1.39, have been used as a dielectric layer because their high optical transparency over a wide wavelength range from 120 nm UV rays in a vacuum to 900 nm infrared rays, as well as good adhesion and high durability.

Recently, it was reported that the light extraction efficiency of GaN-based LEDs could be improved significantly using Al-based ODRs^12^,^13^. On the other hand, such studies did not deal with the influence of the optical properties of LEDs according to the roughness of the dielectric-metal interface in the back-side ODR system. Understanding the detailed mechanism of light interaction in the ODR scheme is important for controlling and maximizing the enhancement of light extraction in LEDs.

This study examined systematically the mechanism of light interaction in the sapphire/MgF_2_/Al triple-layer ODR scheme in terms of the interface roughness with the surface roughness of the sapphire backside. The surface roughness of the sapphire backside critically affected the light extraction efficiency of the ODR scheme and should be minimized to enhance light extraction effectively.

## Results

**Figure 1:**
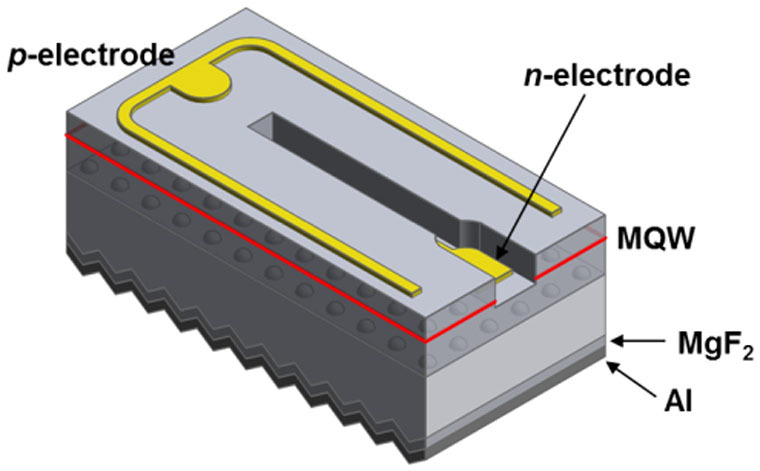
Schematic diagram of a GaN LED with a MgF_2_/Al omnidirectional reflector structure on a roughened sapphire surface.

The AlGaN/InGaN MQW NUV-LED chips were fabricated on c-plane sapphire substrates, and then the sapphire substrate was thinned to 110 μm by backside grinding and lapping using a range of slurries with different diamond sizes of 1, 3 and 5 μm, respectively, to control the surface roughness of the sapphire backside. In addition, chemical mechanical polishing (CMP) was used to make a mirror-like surface at the backside of the sapphire wafer. Subsequently, MgF_2_ (130 nm) and Al (200 nm) layers were deposited conformally on the sapphire backside surface using an e-beam evaporator to form an ODR structure. Finally, the wafer was diced into separate chips by laser-scribing and breakage. Figure 1 shows a schematic diagram of the NUV-LED chip with MgF_2_/Al ODR.

**Figure 2:**
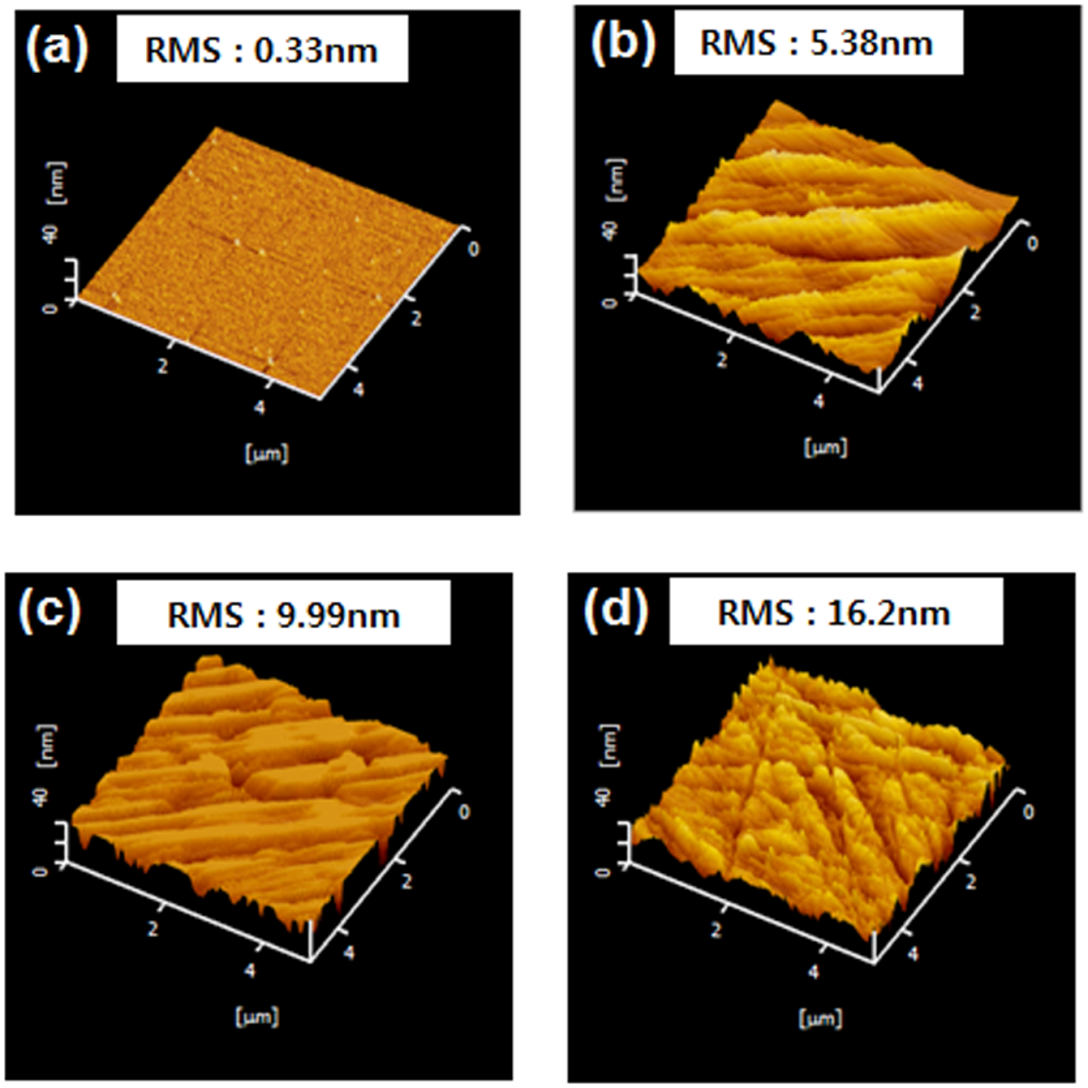
Surface AFM images of the sapphire backsides prepared by (a) CMP, and by lapping in a slurry size of (b) 1 μm, (c) 3 μm, and (d) 5 μm, respectively.

The surface roughness of sapphire backsides prepared by lapping in the slurry with various diamond sizes (1, 3, and 5 μm) (see Supplemental Information for the characteristics of interface roughness and MgF2) and by CMP, was examined by AFM and confocal laser scanning. Figure 2 shows AFM images of the sapphire backsides with a scan area 2 × 2 μm^2^. The root mean square (RMS) roughness was 3.3 Å for CMP, and 53.8 Å (Slurry_1 μm), 99.9 Å (Slurry_3 μm), and 160.2 Å (Slurry_5 μm) for slurry sizes of 1, 3, and 5 μm, respectively. Similarly, the confocal laser scanning of sapphire backsides was performed with a scan area of 125 × 125 μm^2^ as a function of the slurry sizes. The average roughness (R_a_) was 60.0 Å (Slurry_1 μm), 80.0 Å (Slurry_3 μm), and 130.0 Å (Slurry_5 μm) for various slurry sizes. The RMS value is normally higher than that of R_a_, which is the arithmetic average of the absolute values in the roughness profile ordinate and R_a_ is the effective surface roughness measure commonly adopted in general engineering practice. Therefore, this study employed the value R_a_ from confocal laser scanning in the following work.

**Figure 3:**
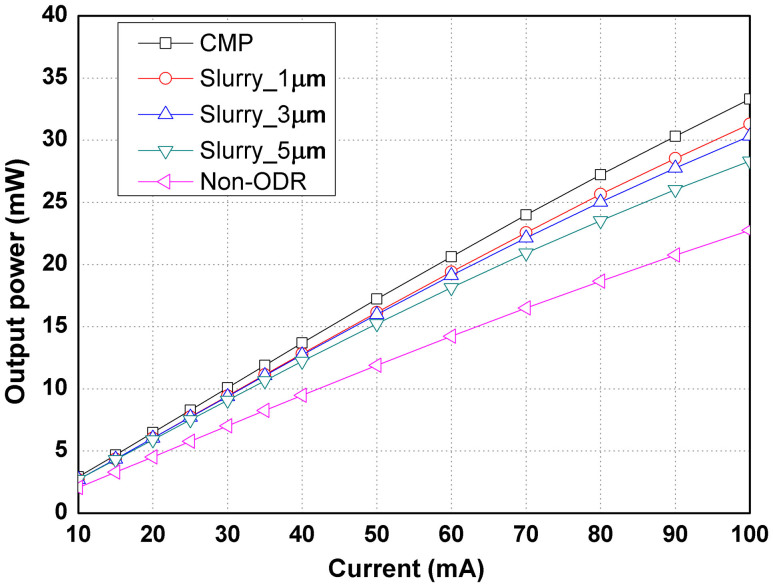
L-I curves of GaN-based NUV-LEDs with and without the MgF_2_/Al ODR structure on various sapphire backsides with different surface roughness.

Figure 3 shows the light output power-current (L-I) curves of GaN-based NUV-LEDs with and without MgF_2_/Al ODR. The light output power of the LEDs with ODR was higher than conventional NUV-LED without ODR. Fig. 3 also shows that the output power of the LEDs with ODR increased with decreasing surface roughness of sapphire with relative enhancements of 21.5% (Slurry_5 μm), 25.5% (Slurry_3 μm), 26.6% (Slurry_1 μm), and 30.9% (CMP), compared to conventional NUV-LED under a 60 mA forward current. It is worthy to note that NUV-LEDs with RMS roughness of 3.3 Å by CMP showed the highest output power (20.6 mW), meanwhile those with RMS roughness of 160.2 Å by slurry size of 5 μm yielded the lowest output power (18.1 mW) under 60 mA forward current among LEDs with ODR structure. These results suggest that the optical output power of GaN-based NUV-LEDs with an ODR structure is influenced significantly by the surface roughness of the sapphire backside.

**Figure 4:**
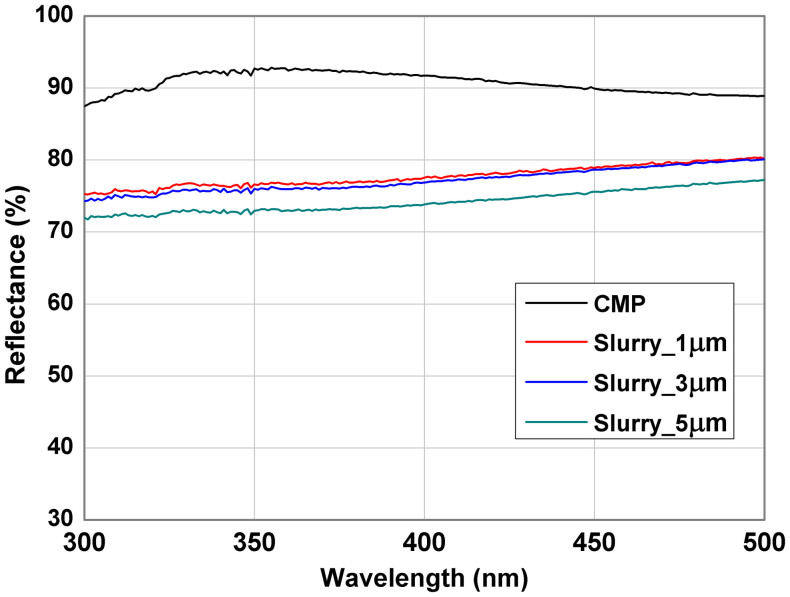
The reflectance vs. wavelength of the MgF_2_/Al ODR structure deposited on various sapphire backsides with different surface roughness.

The reflectance of the MgF_2_/Al ODR structure was measured using a Varian Cary 5000 UV-Vis-NIR through the opposite side of the double-polished sapphire. The integrating sphere system was designed to collect all the diffused photons from a solid surface. Figure 4 shows reflectance of 92.3% (CMP), 76.9% (Slurry_1 μm), 76.3% (Slurry_3 μm), and 73.4% (Slurry_5 μm) at 385 nm in diffuse mode, respectively. These observations agree well with the L-I data trend [Fig. 3] in that the output power of the LEDs and the reflectance of the ODR structure increase with decreasing surface roughness of the sapphire backside. Therefore, the surface roughness of the sapphire backside in the sapphire/MgF_2_/Al ODR structure plays an important role in determining the reflectance of the ODR structure and the light output power of the GaN-based NUV-LEDs.

## Discussion

To understand the observed measurements, we conducted theoretical modeling of the roughened surface of sapphire backside to investigate the light interaction in the sapphire/MgF_2_/Al triple-layer ODR. Firstly, the absorption (Q_ab_) and scattering efficiency (Q_sc_) were calculated to determine the relationship between a single nanoparticle and photon. Secondly, Maxwell-Garnett theory was employed for quantitative analysis in layer-by-layer structures. Lastly, Monte Carlo ray tracing simulation, based upon the results of theoretical modeling, was performed to compare the calculated and measured reflectance of the ODR structure in diffuse mode. In this study, we assumed that the roughened surface of sapphire backside could be considered as a virtual homogeneous layer that is composed of collections of nanoparticles. Normally, the optical and electron energy loss properties of colloids can be modeled reliably by an ordered surface of metal spheres with a filling fraction appropriate to the surface roughness^14^. This would be suitable in an investigation of the optical interaction between a roughened surface and photon. The roughened interfaces of sapphire/MgF_2_ and MgF_2_/Al could be configured as a MgF_2_ particle layer and an Al particle layer, respectively. The Al metallic nanoparticle (MNP) is an intermediate reflector that enhances the near field and effective scattering cross section.

**Figure 5:**
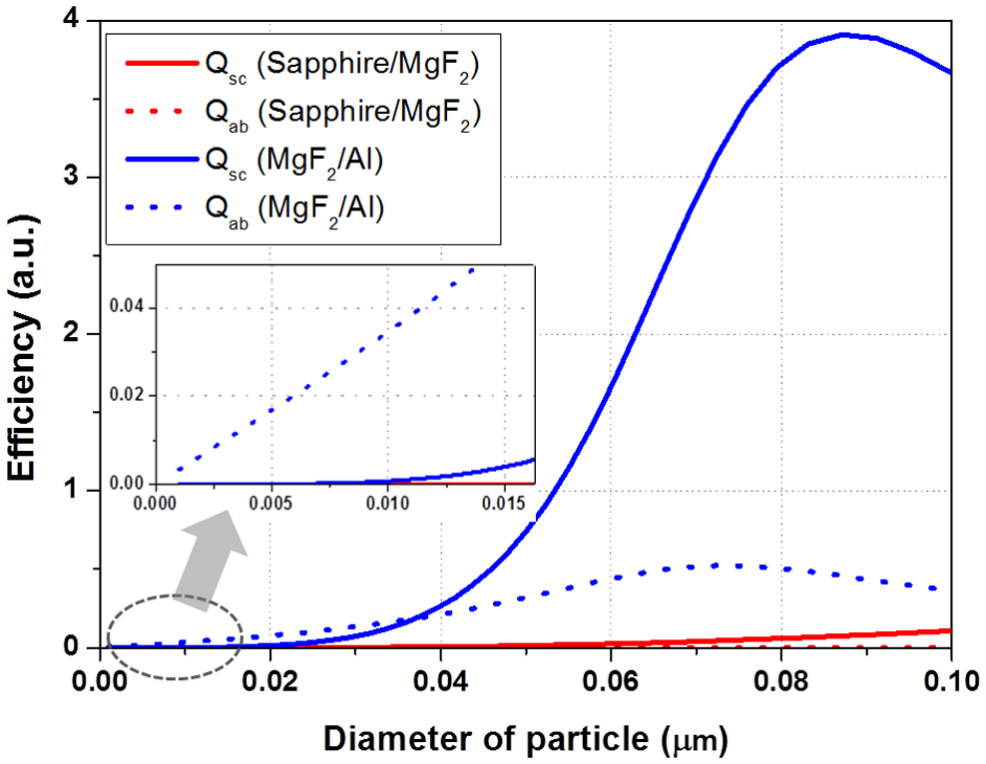
Calculated absorption and scattering efficiencies of MgF_2_ and Al single nanoparticle.

Before the level of nanoparticle array can be approached, the interaction relationship between a single nanoparticle and photon needs to be handled, since it is reasonable to analyze the nano-particle array based on the extension of the interpretation of single nano-particle. In the single MNP model, Q_ab_ and Q_sc_ was calculated using Mie's theory, where Q_ab_ and Q_sc_ are the ratios of their respective total cross section to the physical cross section of the particle. Mie's theory is a general theory of light scattering by a spherical particle^15^,^16^. When the radius r of a particle is much smaller than the wavelength λ of light (i.e. 2πr ≪ λ), the scattering cross-section of a particle varies with r^6^, while the absorption cross-section varies with r^3^. Therefore, for very small particles, absorption is more important than scattering. In addition, when the radius of MNP is much smaller than the incident wavelength, a localized surface plasmon resonance (LSPR) model should be considered further to interpret the interaction between the photon and MNP^17^. The LSPRs are the collective electron charge oscillations in metallic nanoparticles that are excited by light. They exhibit enhanced scattering and absorption at the resonance wavelength. The LSPR mode depends strongly on the extinction characteristics of the MNP structures, such as the dielectric functions of the metal and surrounding materials, size, shape, and density. Figure 5 shows the calculated Q_ab_ and Q_sc_ of MgF_2_ and Al nanoparticles, respectively. The size of R_a_ which generated a major absorption enhancement peak by LSPR mode was approximately 0.07 μm, but it is noteworthy to consider the efficiencies at the tail area with relatively small values. Fig. 5 also shows that absorption and scattering are rarely generated at the interface of sapphire-MgF_2_ in the range of the calculated R_a_ values. On the other hand, the absorption and scattering at the MgF_2_-Al interface increased with increasing particle diameter and the main interaction is the absorption induced by LSPR at the tail area. The scattering effect increased slightly over a 0.01 μm particle diameter. These results can be attributed to that absorption and scattering due to the LSPR do not occur in a dielectric particle since plasma oscillation is rarely generated in the dielectric particle, which result in much smaller absorption and scattering for the MgF_2_ dielectric particle at the sapphire-MgF_2_ interface than the Al metallic particle at the MgF_2_-Al interface, as shown in Fig. 5. This suggests that the absorption and scattering effect at the MgF_2_-Al interface could be related to the light output power of the NUV-LEDs with MgF_2_/Al ODR structures.

**Figure 6:**
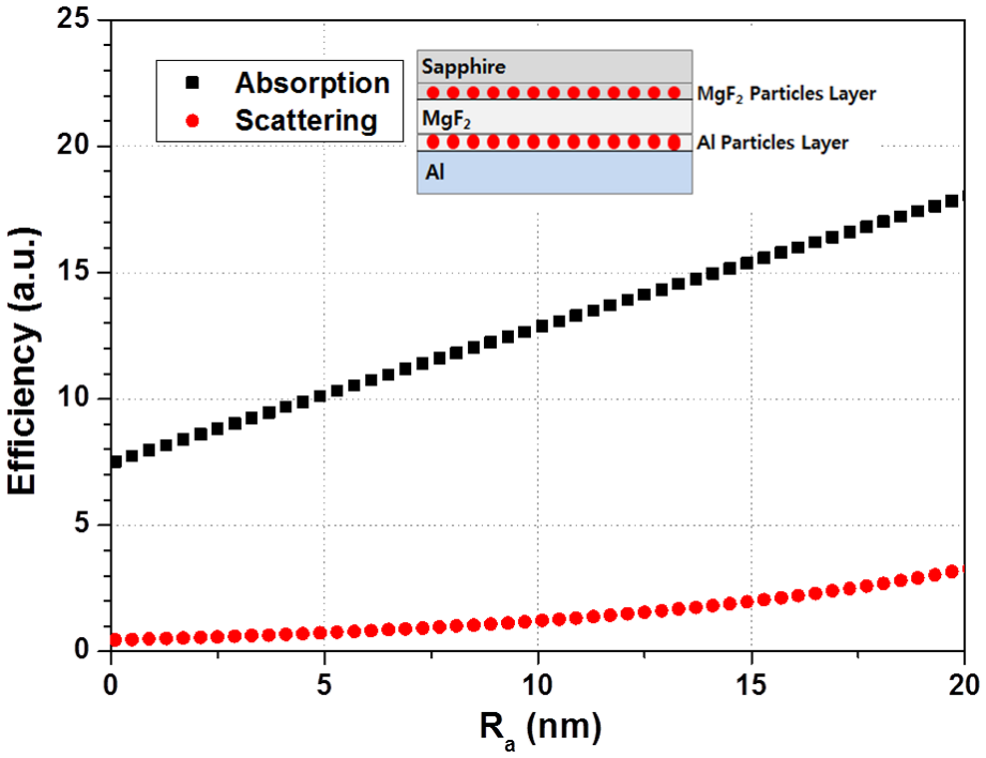
Absorption and scattering efficiency of MgF_2_ and Al particle array as a function of R_a_. The inset shows the 5-layer structure to obtain a correct interpretation of the absorption and scattering efficiency.

Next, the effective refractive indices were calculated based on the effective medium theory (EMT) by Maxwell-Garnett, as extended by Polder and Van Santen to perform quantitative analysis of the optical characteristics^18^,^19^,^20^,^21^,^22^. The Maxwell Garnett approximation is a widely used method for calculating the bulk dielectric properties of inhomogeneous materials. In the effective medium theory, the inhomogeneous material is assumed to be composed of spherical inclusion particles embedded in a host material, both of which are assumed to be isotropic and to respond linearly to the incident light^22^,^23^. A 5-layer structure was implemented to obtain a correct interpretation of the absorption and scattering efficiencies. As shown in the inset in Fig. 6, it was assumed that the sapphire-MgF_2_ interface roughness consisted of a MgF_2_ bulk layer and MgF_2_ particle array, and the MgF_2_-Al interface roughness consisted of an Al bulk layer and Al particle array, which is a good approach for a more correct interpretation of the sapphire-MgF_2_ and MgF_2_-Al interface roughness. The approach could be used to define the optical characteristics for virtual layers, such as the MgF_2_ particle array and Al particle array. The Maxwell-Garnett formula was considered with a volume filling factor (FF), which is defined as the ratio of the volume of occupied particles to the volume with any thickness and the index of the effective medium layer (FF = V_particle_/V_eff_). In addition, the complex optical constants of the virtual layer calculated through EMT were reflected in the Fresnel equations, which were used to define the optical characteristics for each real layer in the simulation. After obtaining the transmittance and reflectance from the Fresnel equation, the extinction coefficient was calculated, after which the extinction coefficient was divided into absorption and scattering. The ratio of scattering and absorption was applied to the ratio obtained from Mie's theory. Eventually, these procedures can reflect the real value in a ray tracing simulation. Figure 6 shows that the efficiency in the 5-layer system is increased tenfold, compared to that in the particle state. In addition, it is shown that as the particle size increases, the scattering and absorption efficiency levels increased with increasing particle size, which is consistent with the results obtained in the case of a single particle model. In Fig. 6, the absorption effect is shown to be the main factor, which generates an approximate nine-fold difference in efficiency, compared to the scattering at R_a_ = 13 nm and the absorption as a function of R_a_ increased linearly to 20 nm, similar to the case of a single particle model. The scattering efficiency increased slightly near R_a_~10 nm, even though the efficiency was much lower than that of absorption.

**Figure 7:**
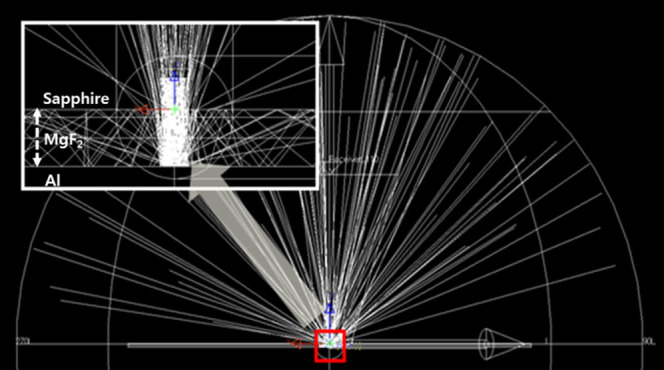
Light propagation simulation image of sapphire/MgF_2_/Al triple-layer ODR structures. The inset is a magnified image showing the internal reflection.

**Figure 8:**
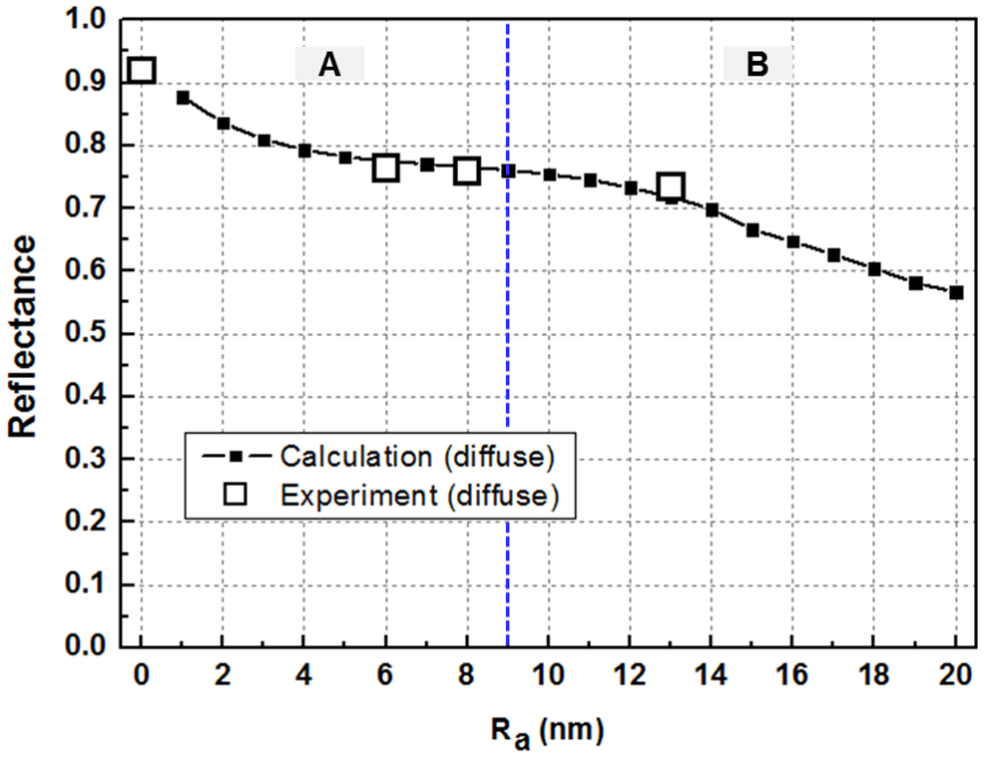
Calculated reflectance and the experimental reflectance for the MgF_2_/Al ODR structure as a function of R_a_ in the diffuse mode.

Finally, ray tracing simulation, based upon the results of theoretical modeling, was performed to compare the calculated and measured reflectance of the ODR. In this study, the Monte Carlo ray-tracing method, which is a representative way of simulating light propagation in LED chips was used to examine the light propagation behavior of sapphire/MgF_2_/Al triple-layer ODR structures. Figure 7 shows that a significant amount of photons can be confined between the sapphire and Al metal layer by a scattering process at the roughened interfaces and be guided in the MgF_2_ layer, resulting in the increased absorption of trapped photons by the continuous internal reflection. Note that the amount of internal reflection is not negligible. The increased absorption by internal reflection in the ODR structure will reduce the light extraction efficiency of the LEDs with ODRs. Therefore, the backside surface roughness of sapphire critically influences the light extraction efficiency of LEDs with the ODRs through scattering at the rough interface. Figure 8 presents the calculated and measured reflectance for the sapphire/MgF_2_/Al ODR structure as a function of R_a_ in diffuse mode. As shown in Figs. 5 and 6, the absorption efficiency increased almost linearly with increasing particle size in the single nanoparticle model as well as the 5-layer model, indicating a linear decrease in reflectance with increasing size. On the other hand, the measured reflectance did not decrease linearly with increasing R_a_. This discrepancy could be solved by considering the volume filling factor (FF) in the Maxwell-Garnett formula mentioned above. The calculated reflectance by ray tracing with the consideration of the FF showed good agreement with the measured reflectance in diffuse mode, as shown in Fig. 8. The calculated and measured reflectance decreased nonlinearly with increasing R_a_. The nonlinear decrease in reflectance with R_a_ could be explained as follows. The nonlinearity can be divided into two sections; section A with a FF-dependent zone of R_a_ ranging from 0 to 9 nm, which shows a rapid decrease in the reflectance with R_a_, and section B with a size-dependent zone in the range of R_a_ over 9 nm. The FF value of section A was larger than that of section B, which is because the FF value increases with decreasing R_a_. Although the individual particle size is small in section A, the density (FF) is increased drastically, so the absorption effect is increased. The FF value of section B is relatively constant, but the individual particle size is increased, which means that the scattering effect plays an important role in section B. As a result, absorption and scattering effects occur at the same time in section B. Therefore, the optical output power of NUV-LEDs with MgF_2_/Al ODR structures decrease gradually with increasing sapphire backside roughness, which was attributed to the absorption effect induced by LSPR and photon confinement in MgF_2_ by the scattering effect.

In summary, we have investigated the mechanism of light interaction in the sapphire/MgF_2_/Al triple-layer ODR and its effects on the light output power in NUV-LEDs with the ODR structures deposited on the back-side surface of sapphire substrates with various surface roughness. The light output power of the NUV-LED with ODR deposited on the mirror-like surface of sapphire was improved by 30.9% compared to the conventional LED without ODR. The surface roughness of the sapphire backside in the MgF_2_/Al ODR structure played an important role in determining the reflectance of the ODR structure and the light output power of the LEDs. The dependence of the reflectance of the ODR structure on the surface roughness was attributed mainly to the absorption effect induced by LSPR and photon confinement in MgF_2_ due to the scattering effect.

## Methods

### Fabrication

The NUV LED structures were grown on c-plane sapphire substrates by metal-organic chemical vapor deposition. Ammonia, trimethylgallium, trimethylaluminium, and trimethylindium were used as the precursors and biscyclopen-tadienilmagnesium and silane were used as the dopants. The epitaxial NUV-LED structure consisted of a low-temperature 30-nm-thick GaN buffer layer, 4-μm-thick *n*-type GaN layer, six pairs of 6-nm-thick Al_0.1_GaN/3-nm-thick In_0.03_GaN multiple quantum wells, a 25-nm-thick *p*-type Al_0.25_GaN electron-blocking-layer to prevent carrier leakage from the active region, and a 0.2-μm-thick *p*-type GaN layer. The InGaN active layer was grown at 820°C under 200 Torr with a growth rate of 1 nm/min. The thicknesses and compositions in the AlGaN/InGaN MQWs were determined by x-ray diffraction (XRD) using a Cu K_α_ x-ray source. An in-situ optical reflectometer was employed to monitor the morphological evolution and growth rates of epitaxial layers. After growth, thermal annealing was performed for 15 min at 725°C in a nitrogen atmosphere to obtain *p*-type conductivity of Mg-doped GaN. The AlGaN/InGaN MQW NUV-LED chips were then fabricated. All samples were immersed into H_2_SO_4_:H_2_O_2_ solution for 10 min to remove metallic contaminants on the surface and then rinsed in running deionized water. The mesa structure for the 700 × 300 μm^2^ chip was formed partially until the *n*-GaN contact layer was exposed using an inductively coupled plasma etching system in conjunction with Cl_2_ and a standard photolithography process. The thin film of indium-tin-oxide for the *p*-ohmic metal was deposited as a transparent conducting layer by sputter deposition and alloyed in N_2_ ambient for 60 s at 500°C to reduce the contact resistance. Wet etch of ITO was achieved by using HCl aqueous solution with photoresist as an etch mask. Subsequently, Ti/Al/Ni/Au films were deposited using an electron-beam evaporator for use as the *p*- and *n*-type contact metals, and a 0.12-μm-thick SiO_2_ layer was deposited by plasma enhanced chemical vapor deposition to passivate the mesa side wall. The sapphire substrate was thinned to 110 μm by backside grinding and lapping using a range of slurries with different diamond sizes of 1, 3 and 5 μm, respectively, to control the surface roughness of the sapphire backside. In addition, chemical mechanical polishing (CMP) was used to make a mirror-like surface at the backside of the sapphire wafer after grinding and lapping to about 120 μm. MgF_2_ (130 nm) and Al (200 nm) layers were subsequently deposited on the sapphire backside surface using an e-beam evaporator at room temperature to form an ODR structure. Finally, the wafer was diced into separate chips by laser-scribing and breakage.

### Characterization

The reflectance on the sapphire/MgF_2_/Al triple-layer ODR structure was measured using a Varian Cary 5000 UV-Vis-NIR system. A Xenon lamp covering the entire wavelength range of 190–1100 nm was used as the incident light source. The current-voltage characteristics of the NUV-LEDs were analyzed at room temperature using a Keithley 2601A parameter analyzer, and the optical output power characteristics of the NUV-LEDs with FR4-PCB were analyzed using an integrated sphere with a calibrated power meter. The surface roughness of sapphire backsides prepared by lapping in the slurry with various diamond sizes (1, 3, and 5 μm) and by CMP, was examined by AFM and confocal laser scanning.

## Additional Information

**How to cite this article**: Lee, K.H., Moon, Y.-T., Song, J.-O. & Kwak, J.S. Light interaction in sapphire/MgF_2_/Al triple-layer omnidirectional reflectors in AlGaN-based near ultraviolet light-emitting diodes. *Sci. Rep.* 5, 9717; DOI:10.1038/srep09717 (2015).

## Supplementary Material

Supplementary Information Supplementary Information

## References

[b1] Khan A., Balakrishnan K. & Katona T. Ultraviolet light-emitting diodes based on group three nitrides. Nature Photon. 2, 77–84 (2008).

[b2] Pimputkar S., Speck J. S., DenBaars S. P. & Nakamura S. Prospects for LED lighting. Nature Photon. 3, 180–182 (2009).

[b3] Kim J. S., Jeon P. E., Choi J. C., Park H. L., Mho S. I. & Kim G. C. Warm-white-light emitting diode utilizing a single-phase full-color Ba_3_MgSi_2_O_8_:Eu^2+^, Mn^2+^ phosphor. Appl. Phys. Lett. 84, 2931 (2004).

[b4] Sohn K. S., Park D. H., Cho S. H., Kwak J. S. & Kim J. S. Computational evolutionary optimization of red phosphor for use in tricolor white LEDs. Chem. Mater. 18, 1768–1772 (2006).

[b5] Kim J. S. *et al.* White-light generation through ultraviolet-emitting diode and white-emitting phosphor. Appl. Phys. Lett. 85, 3696 (2004).

[b6] Lee J. S., Lee J., Kim S. & Jeon H. GaN light-emitting diode with deep-angled mesa sidewalls for enhanced light emission in the surface-normal direction. IEEE Trans. Electron. Dev. 55, 523–526 (2008).

[b7] Oder T. N., Shakya J., Lin J. Y. & Jiang H. X. III-nitride photonic crystals. Appl. Phys. Lett. 83, 1231–1233 (2003).

[b8] Zhao Y. S., Hibbard D. L., Lee H. P., Ma K., So W. & Liu H. Efficiency enhancement of InGaN/GaN light-emitting diodes with a back-surface distributed Bragg reflector. J. Electron. Mater. 32, 1523–1526 (2003).

[b9] Kim J. K. *et al.* Enhanced light-extraction in GaInN near-ultraviolet light-emitting diode with Al-based omnidirectional reflector having NiZn/Ag microcontacts. Appl. Phys. Lett. 89, 141123 (2006).

[b10] Huang K., Gao N., Wang C., Chen X., Li J., Li S., Yang X. & Kang J. Top- and bottom-emission-enhanced electroluminescence of deep-UV light-emitting diodes induced by localized surface plasmons. Sci. Rep. 4, 4380 (2014). 24625660 10.1038/srep04380 PMC3953721

[b11] Gao N., Huang K., Li J., Li S., Yang X. & Kang J. Surface-plasmon-enhanced deep-UV light emitting diodes based on AlGaN multi-quantum wells. Sci. Rep. 2, 816 (2012). 23150780 10.1038/srep00816 PMC3495303

[b12] Kim H. S., Lee S. N., Park Y. J., Kim K. K., Kwak J. S. & Seong T. Y. Light extraction enhancement of GaN-based light emitting diodes using MgF_2_/Al omnidirectional reflectors. J. Appl. Phys. 104, 053111 (2008).

[b13] Kim J. K. *et al.* Enhanced light-extraction in GaInN near-ultraviolet light-emitting diode with Al-based omnidirectional reflector having NiZn/Ag microcontacts. Appl. Phys. Lett. 89, 141123 (2006).

[b14] Garcia-Vidal F. J. & Pendry J. B. Electromagnetic interactions with rough metal surfaces. Progress in Surf. Sci. 50, 55–64 (1995).

[b15] Mie G. Beiträge zur Optik trüber Medien. Ann. d. Physik 25, 377–445 (1908).

[b16] van de Hulst H. C. Light Scattering by Small Particles, Ch. 9, 114–130 (Dover, New York, 1981).

[b17] Zeng S., Yong K. T., Roy I., Dinh X. Q., Yu X. & Luan F. A review on functionalized gold nanoparticles for biosensing applications. Plasmonics 6, 491–506 (2011).

[b18] Kreibig U. & Vollmer M. Optical Properties of Metal Clusters, Ch. 2, 12–201 (Springer Series in Materials Science Vol. 25, Berlin, 1995).

[b19] Bohren C. F. & Huffman D. R. Absorption and Scattering of Light by Small Particles, Ch. 8, 214–219 (Wiley, New York, 1983).

[b20] Granqvist C. G. & Hunderi O. Optical properties of ultrafine gold particles, *Phys*. Rev. B 16, 3513–3534 (1977).

[b21] Xu G., Tazawa M., Jin P., Nakao S. & Yoshimura K. Wavelength tuning of surface plasmon resonance using dielectric layers on silver island films. Appl. Phys. Lett. 82, 3811–3813 (2003).

[b22] Maxwell Garnett J. C. Colours in Metal Glasses and in Metallic Films. Philos. Trans. R. Soc. London, 203, 385 (1904).

[b23] Sipe J. E. & Boyd R. W. Nonlinear susceptibility of composite optical materials in the Maxwell Garnett model. Phys. Rev. A 46, 1614 (1992). 9908285 10.1103/physreva.46.1614

