# Supplementary material for: Light interaction in sapphire/MgF2/Al triple-layer omnidirectional reflectors in AlGaN-based near ultraviolet light-emitting diodes
Source: Sci Rep. 2015 May 26;5:9717. doi: 10.1038/srep09717 (PMC4443774; doi:10.1038/srep09717)
Supplement: Supplementary Information [file srep09717-s1.doc]

Light interaction in sapphire/MgF_2_/Al triple-layer omnidirectional reflectors in AlGaN-based near ultraviolet light-emitting diodes

Keon Hwa Lee^1^, Yong-Tae Moon^1,3^, June-O Song^1^, and Joon Seop Kwak^2,*^

^1^ Department of LED Business, LG Innotek Company, Ltd., Paju, Korea

^2^ Department of Printed Electronics Engineering, Sunchon National University, Jeonnam, Korea

^3^ ytmoon@lginnotek.com, ^*^jskwak@sunchon.ac.kr

**Characterization of the MgF_2_/sapphire interface with cross-sectional transmission electron microscopy (XTEM)**

We have characterized the MgF_2_/sapphire interface by using XTEM, and the results are as follow. We analyzed the sapphire-MgF_2_ interface, where the sapphire backside was prepared by chemical-mechanical polishing (CMP). We found that the interface between MgF_2_ and CMP-sapphire backside was smooth, which implies that CMP-finished sapphire backside yielded a smooth surface as expected in AFM analysis shown in Fig. 2 in the manuscript (RMS roughness of 3.3Å) and electron-beam evaporation of MgF_2_ on the CMP-finished sapphire backside produced a smooth interface.


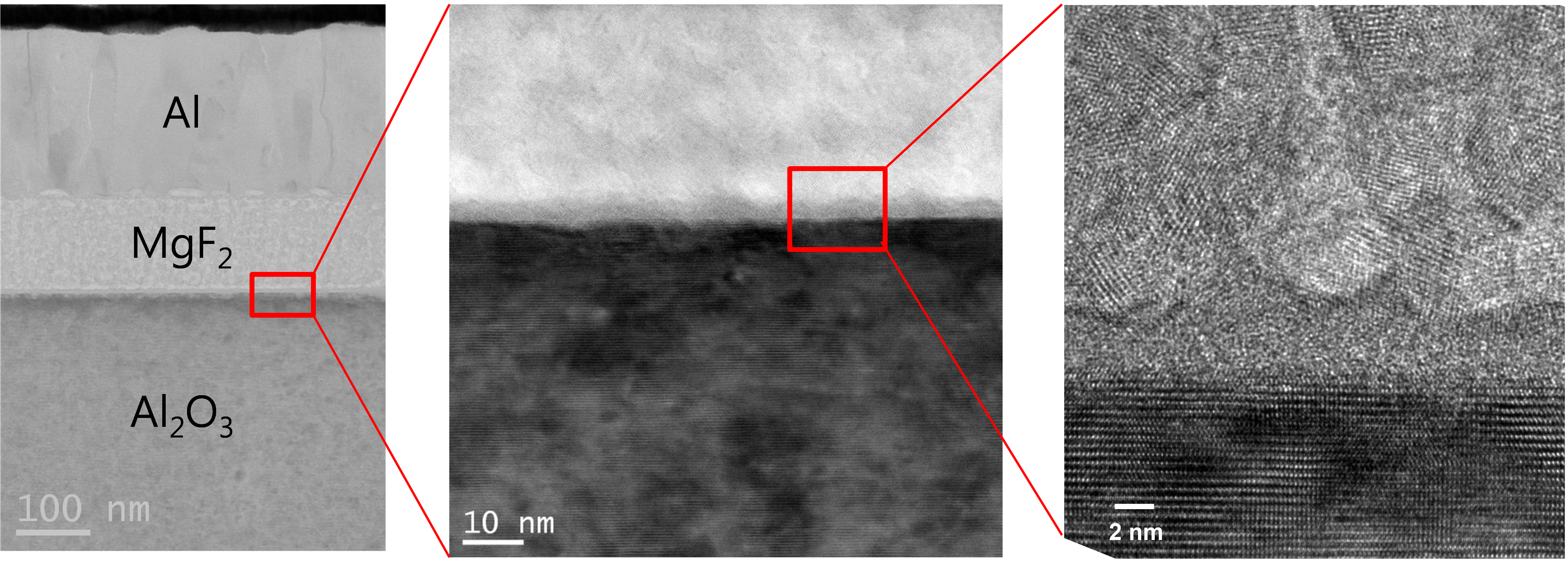


**Characterization of the crystalline structure of MgF_2_ with XTEM**

We also investigated the crystalline structure of MgF_2_ by using XTEM, and the results are as follow. We found that MgF_2_ layer showed nano-crystalline grain structures with a diameter of ~ 10 nm. We also found that the crystalline structure of MgF_2_ at the MgF_2_-CMP sapphire backside interface was similar to that of MgF_2_ at the region near Al layer, which implies that MgF_2_ has a uniform crystalline structure throughout the MgF_2_ layer.
